# Sam68 reduces cisplatin-induced apoptosis in tongue carcinoma

**DOI:** 10.1186/s13046-016-0390-3

**Published:** 2016-07-29

**Authors:** Shuwei Chen, Huan Li, Shimin Zhuang, Ji Zhang, Fan Gao, Xidi Wang, WenKuan Chen, Ming Song

**Affiliations:** 1 State Key Laboratory of Oncology in South China, Collaborative Innovation Center of Cancer Medicine, Guangzhou, China; 2 Department of Head and Neck Surgery, Sun Yat-Sen University Cancer Center, Guangzhou, China; 3 Department of Intensive Care Unit, Sun Yat-Sen University Cancer Center, Guangzhou, China; 4 Department of Otolaryngology-Head and Neck Surgery, The Sixth Affiliated Hospital of Sun Yat-Sen University, Guangzhou, China

**Keywords:** Sam68, Tongue squamous cell carcinoma, Anti-apoptosis, Chemotherapy

## Abstract

**Background:**

Resistance to anticancer agents is a major obstacle for successful chemotherapy in tongue squamous cancer. Sam68 is an oncogenic-related protein in oral tongue squamous cell carcinoma functions as a signaling molecule mediating apoptosis, whose over-expression is associated with the clinicopathologic characteristics and prognosis of patients. The present study was to examine the effect of Sam68 on chemotherapeutics-induced apoptosis in oral tongue squamous cell carcinoma, and its clinical significance in oral tongue squamous cell carcinoma progression.

**Methods:**

The effect of Sam68 on apoptosis induced by cisplatin was examined both in vitro and in vivo, using Annexin V staining and terminal deoxynucleotidyl transferase-mediated dUTP nick end labeling assays. Real-time PCR and Western blotting analysis were used to detect mRNA and protein expression levels.

**Results:**

Upregulation of Sam68 significantly inhibited cisplatin-induced apoptosis in oral tongue squamous cell carcinoma cells, associated with induction of anti-apoptotic proteins caspase-9, caspase-3, and PARP. In contrast, Silencing Sam68 expression significantly enhanced the sensitivity of oral tongue squamous cell carcinoma cells to apoptosis induced by cisplatin both in vitro and in vivo.

**Conclusions:**

The current study suggests that Sam68 could enhance the anti-apoptosis activity of oral tongue squamous cell carcinoma cells. Sam68 is a potential pharmacologic target for the treatment of oral tongue squamous cell carcinoma and inhibition of Sam68 expression might represent a novel strategy to sensitize oral tongue squamous cell carcinoma to chemotherapy.

## Background

Worldwide, approximately 263,900 cases of oral cancer are diagnosed each year, making oral cancer the 10th most commonly diagnosed cancer in men. The most common pathological type of oral tongue cancer is squamous cell carcinoma [1]. The tongue is the most cancer-prone intra-oral site in most populations [2]. Despite advances in standard treatment strategies (e.g., surgery, chemotherapy, and radiotherapy), the mortality rate in patients with OTSCC has remained largely unchanged for decades, with a 5-year survival rate of approximately 50 % [3]. The 5-year survival rate for patients with tongue cancer is 78 % for cancer exhibiting local spread, 63 % for cancer exhibiting regional spread, and 36 % for cancer exhibiting distant spread according to the American Cancer Society [4, 5].

Cisplatin-based chemotherapy (DDP), as a common standard therapeutic approach, plays an important role in tongue cancer treatment and results in many therapeutic benefits, including reducing tumor size, inhibiting the formation of distant metastatic lesions, and prolonging patient survival [6]. Resistance to anticancer agents is a major challenge for achieving successful chemotherapy in tongue cancer and can result in more aggressive tumor behavior and poor clinical outcomes [7, 8]. The molecular basis of resistance to chemotherapy is complex and involves various biological processes, such as drug transport, drug metabolism, apoptosis, and DNA repair [9]. Although the mechanisms of resistance in cancer have been extensively studied for decades, little is known about the clinical causes of drug resistance. In order to overcome drug resistance in tongue cancer treatment, it will be necessary to clarify the mechanisms of drug resistance and explore alternative therapeutic strategies.

Src-associated protein in mitosis (Sam68, 68 kDa), a member of the signal transduction and activation of RNA (STAR) family, has been identified as an oncogenic protein [10]. The function of Sam68 can be attributed to its RNA-binding properties, through which Sam68 regulates metabolism, nuclear export, and RNA stability. Emerging evidence has suggested that Sam68 functions as a signaling molecule in multiple signaling pathways [11]. In particular, a recent study showed that Sam68 functions in both nuclear factor kappaB (NF-kB) activation and apoptosis initiated through the tumor necrosis factor (TNF) receptor [12]. In a previous study, we found that Sam68 is overexpressed in OTSCC and is significantly associated with the clinicopathological characteristics and prognosis of patients [13]. Nonetheless, the relationship between Sam68 overexpression and the biological behaviors of human OTSCC remain unexplored.

Therefore, in this study, we investigated the relationship between Sam68 status and responses to chemotherapy both in vitro and in vivo and evaluated the predictive value of Sam68 expression in OTSCC cell lines treated with the DNA-damaging chemotherapeutic drug cisplatin.

## Methods

### Cell lines

Two human OTSCC cell lines, i.e., SCC-9 and SCC-25, were obtained from the American Type Culture Collection (ATCC, Manassas, VA, USA). Cells were cultured in Dulbecco’s modified Eagle medium (Invitrogen, Carlsbad, CA, USA) supplemented with 10 % fetal bovine serum (HyClone). Cells were grown in a 5 % CO_2_ humidified atmosphere at 37 °C.

### Plasmids and transfection

A Sam68 overexpression construct was generated by subcloning of polymerase chain reaction (PCR)-amplified full-length human Sam68 cDNA into the pMSCV plasmid. For depletion of Sam68, two human Sam68-targeting siRNA sequences were cloned into the pSuper-retro-puro plasmid to generate pSuper-retro-Sam68-RNAi(s). The sequences were as follows (synthesized by Invitrogen): RNAi#1: GGACCACAAGGGAATACAATC; RNAi#2: GCATCCAGAGGATACCTTTGC (5′ to 3′). Retroviral production and infection were performed as described previously [14]. Stable cell lines expressing Sam68 or Sam68 shRNAs were selected for 10 days with 0.5 μg/mL puromycin.

### RNA extraction, reverse transcription, and real-time PCR

Total RNA was extracted from cultured cells using TRIzol reagent (Invitrogen) according to the manufacturer’s instructions. cDNAs were amplified and quantified using an ABI Prism 7500 Sequence Detection System (Applied Biosystems, Foster City, CA, USA) with SYBR Green I dye (Molecular Probes, Eugene, OR, USA). Expression data were normalized to the geometric mean of the housekeeping gene β-actin. The primers were selected as follows:

Sam68, forward 5′-ATGAAGCTTATGGCCAGGAC-3′ and reverse 5′-CAGAAGCCAGAATGCAGAGT-3′, β-actin, forward 5′-TGGCACCCAGCACAATGAA-3′ and reverse 5′-CTAAGTCATAGTCCGCCTAGAAGCA-3′.

Real-time PCR was performed according to standard methods, as described previously [13].

### Western blotting

Western blotting was performed according to standard methods, as described previously,^12^ using polyclonal rabbit anti-Sam68 antibodies (sc-733; Santa Cruz Biotechnology, Santa Cruz, CA, USA), anti-caspase-9, anti-cleaved caspase-9, anti-caspase-3, anti-cleaved caspase-3, anti-poly (ADP-ribose) polymerase (PARP), and anti-cleaved PARP antibodies (Cell Signaling Technology, Danvers, MA, USA). The membranes were stripped and reprobed with an anti-β-actin antibody (Sigma, St. Louis, MO, USA) as a loading control.

### Cell growth assay

The cell growth rates of cells with different levels of Sam68 expression were determined by 2,3-bis-(2-methoxy-4-nitro-5-sulfophenyl)-2H-tetrazolium-5-car-boxanilide(XTT) assays. Cells were seeded into 96-well plates at a density of 1 × 10^3^ cells/well. The XTT kit (Sigma) was used according to the manufacturer’s instruction. Triplicate independent experiments were performed.

### Terminal deoxynucleotidyl transferase-mediated dUTP nick end labeling (TUNEL) assays

The DeadEnd Fluorometric TUNEL system (Promega, Madison, WI, USA) was used for TUNEL assays according to the manufacturer’s instructions. A total of 3 × 10^4^ cells were seeded on coverslips (Fisher Scientific) in 24-well plates. After 24 h, all cells were incubated with cisplatin, washed once with cold phosphate-buffered saline (PBS), and fixed in freshly prepared 4 % formaldehyde solution in PBS (pH 7.4) for 25 min at 4 °C. The fixed slides were washed with PBS for 5 min and then permeabilized with 0.2 % Triton X-100 solution in PBS for 5 min. After a 5-min wash with PBS, and cells were covered with 100 μL Equilibration Buffer for 5 min. Cells were then incubated for 60 min at 37 °C to terminate the reaction and then washed in PBS for 5 min. The samples were then stained in the dark with 1 μg/mL propidium iodide (PI) solution for 15 min. After a final wash with H_2_O for 5 min at ambient temperature and air drying, samples were immediately analyzed under a fluorescence microscope using a standard fluorescein filter set to view the green fluorescence of fluorescein at 520 nm and the red fluorescence of PI at 620 nm.

### Annexin-V binding assay

An ApopNexin FITC Apoptosis Detection Kit (Millipore, Lake Placid, NY, USA) was used to examine apoptotic cells according to the manufacturer’s instructions. A total of 3 × 10^5^ cells were seeded in triplicate in 6-well plates. After 24 h, all cells were incubation with cisplatin, followed by washes with PBS and then the Annexin-V binding solution. Subsequently, 150 μL of the Annexin-V antibody in Binding Buffer was added to each culture well. Cells were incubated for 15 min, 1.5 μL of PI was added at 1 mg/mL, and cells were further incubated for 5 min. A total of 10,000 cells were analyzed on a flow cytometer (FACSCalibur; BD Biosciences).

### Xenograft tumor model in vivo

Female BALB/c nude mice (4–5 weeks of age, 18–20 g) were purchased from the Center of Experimental Animals of Guangzhou University of Chinese Medicine and were housed in barrier facilities on a 12-h light/dark cycle. All animal work was performed under the approval of the Institutional Animal Care and Use Committee of Sun Yat-sen University. BALB/c nude mice were randomly divided into four groups (*n* = 10/group). For tumor cell implantation, two groups of the mice were inoculated subcutaneously with the SCC-9/vector and SCC-9/scramble cells (1 × 10^6^ per mouse, suspended in 100 μL sterile PBS) in the right oxter as control groups. The other two groups were inoculated with SCC-9/Sam68 and SCC-9/Sam68 RNAi#1 cells (1 × 10^6^ per mouse, suspended in 100 μL sterile PBS). Tumors were examined twice weekly; length, width, and thickness measurements were obtained with calipers, and tumor volumes were calculated using the equation (L × W^2^)/2. When tumors reached a volume of 50–100 mm^3^, each group was randomly assigned to two subgroups (*n* = 5/group), followed by intraperitoneal injection of 100 μL PBS or cisplatin (2.5 mg/kg) on days 0, 3, 7, 10, 14, 17, 21, and 24. On day 28, animals were euthanized, and tumors were excised, weighed, and subjected to pathological examination.

### Statistical analysis

All statistical analyses were carried out using the SPSS 17.0 statistical software package. Comparisons between groups for statistical significance were performed with a two-tailed paired Student’s *t* test. The *χ*^2^ test was used to analyze the relationship between Sam68 expression and clinicopathological characteristics. Survival curves were plotted using the Kaplan-Meier method and compared by the log-rank test. Survival data were evaluated using univariate and multivariate Cox regression analyses. Differences with P values of less than 0.05 were considered statistically significant in all cases.

## Results

### Constructing stable Sam68 expression cell lines

**Fig. 1:**
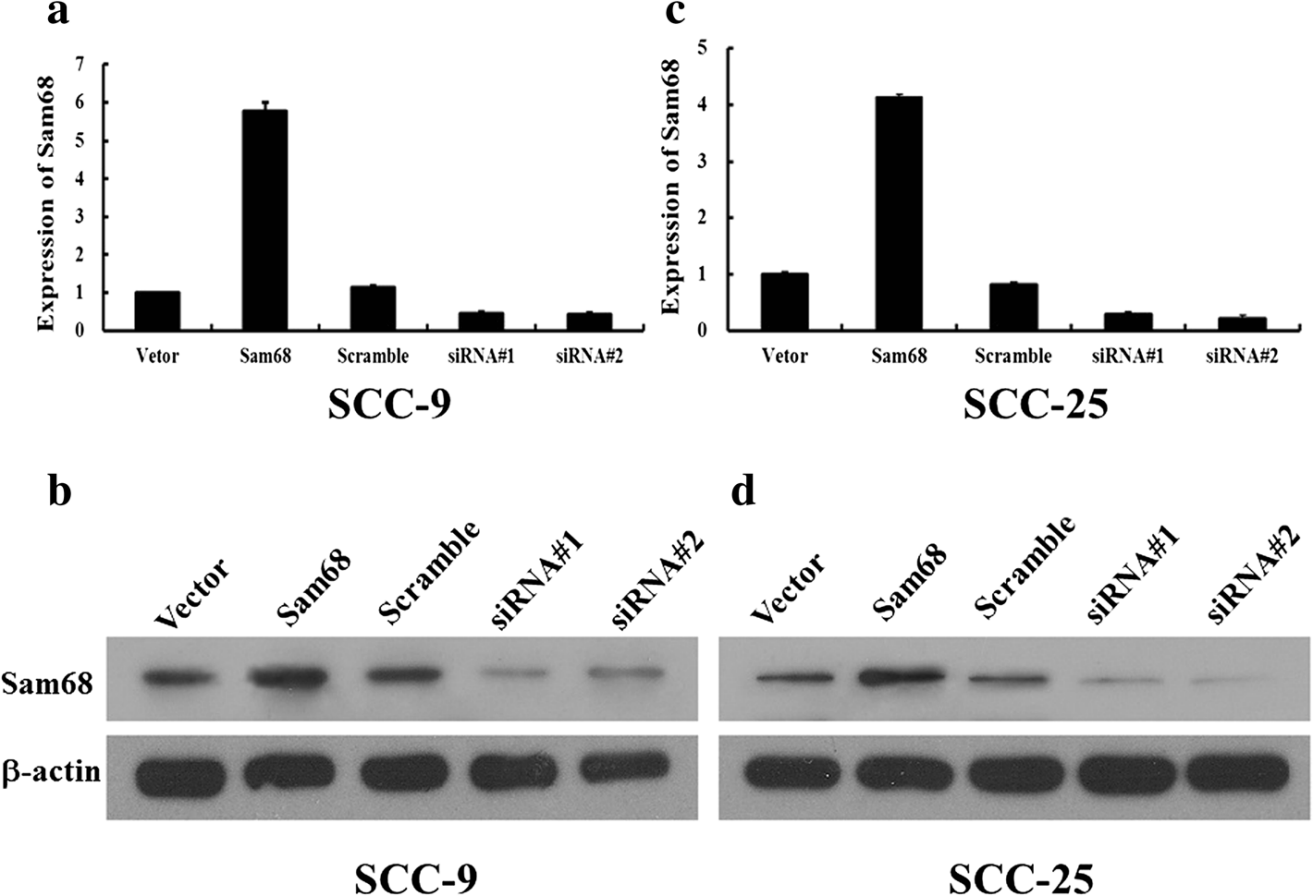
RT-PCR (**a**, **c**) and Western blotting (**b**, **d**) showed that Sam68 was highly expressed in Sam68-overexpressing cells relative to that in scramble and blank vector control cells after 4 days of culture. Conversely, Sam68 was downregulated in Sam68-silenced cells relative to that in scramble and blank vector control cells after 4 days of culture. β-actin expression was used as control

Two OTSCC cell lines, SCC-9 and SCC-25, were constructed to stably overexpress Sam68, producing SCC-9/Sam68 and SCC-25/Sam68 cells, or to stably silence Sam68, producing SCC-9/shSam68 and SCC-25/shSam68 cells. SCC-9/vector, SCC-9/scramble, SCC-25/vector, and SCC-25/scramble were used as control cells. Western blotting and reverse transcription (RT)-PCR were used to test Sam68 expression (Fig. 1). The results showed that Sam68 was highly expressed in Sam68-overexpressing cells relative to that in scramble and blank vector control cells after 4 days of culture. Conversely, Sam68 was downregulated in Sam68-silenced cells relative to that in scramble and blank vector control cells after 4 days of culture. As a control, β-actin expression was not altered.

### Dysregulation of Sam68 altered apoptosis in OTSCC cells

**Fig. 2:**
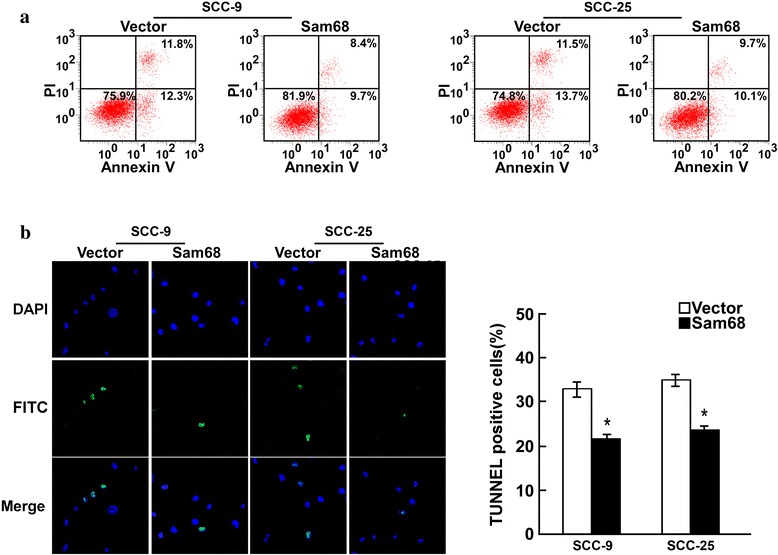
Annexin V-binding (**a**) and TUNEL assays (**b**) showed that Sam68-overexpressing SCC-9 and SCC-25 cells exhibited significantly higher survival rates than vector-control cells cultured under the same conditions

**Fig. 3:**
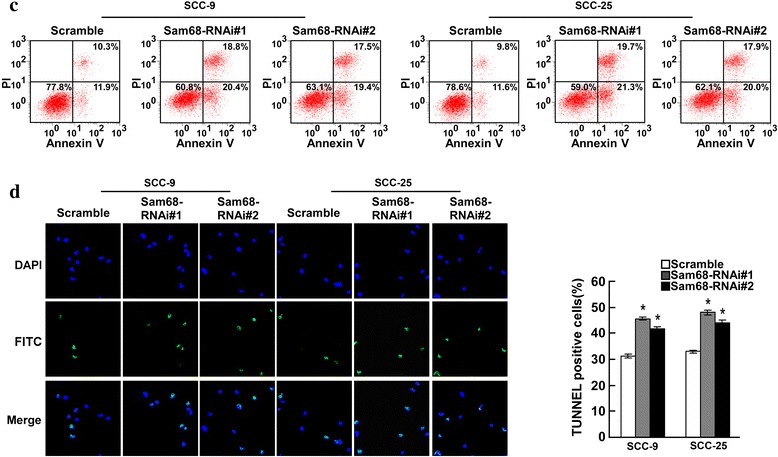
Annexin V-binding(**c**) and TUNEL assays (**d**) showed that Sam68 silenced SCC-9 and SCC-25 cells exhibited significantly lower survival rates than Scramble cells cultured under the same conditions. But the survival rates between two samples of SCC-9 or SCC-25 had little difference

To further elucidate and characterize the anti-apoptotic activity of Sam68 in OTSCC cells, in vitro studies were performed using OTSCC cell lines with overexpression or silencing of Sam68. Annexin V-binding and TUNEL assays showed that Sam68-overexpressing SCC-9 and SCC-25 cells exhibited significantly higher survival rates than vector-control cells cultured under the same conditions (Fig. 2). In contrast, the number of dead cells markedly increased when Sam68 expression was silenced by specific shRNA (Fig. 3).

### Dysregulation of Sam68 altered the chemosensitivity of OTSCC cells in vitro

**Fig. 4:**
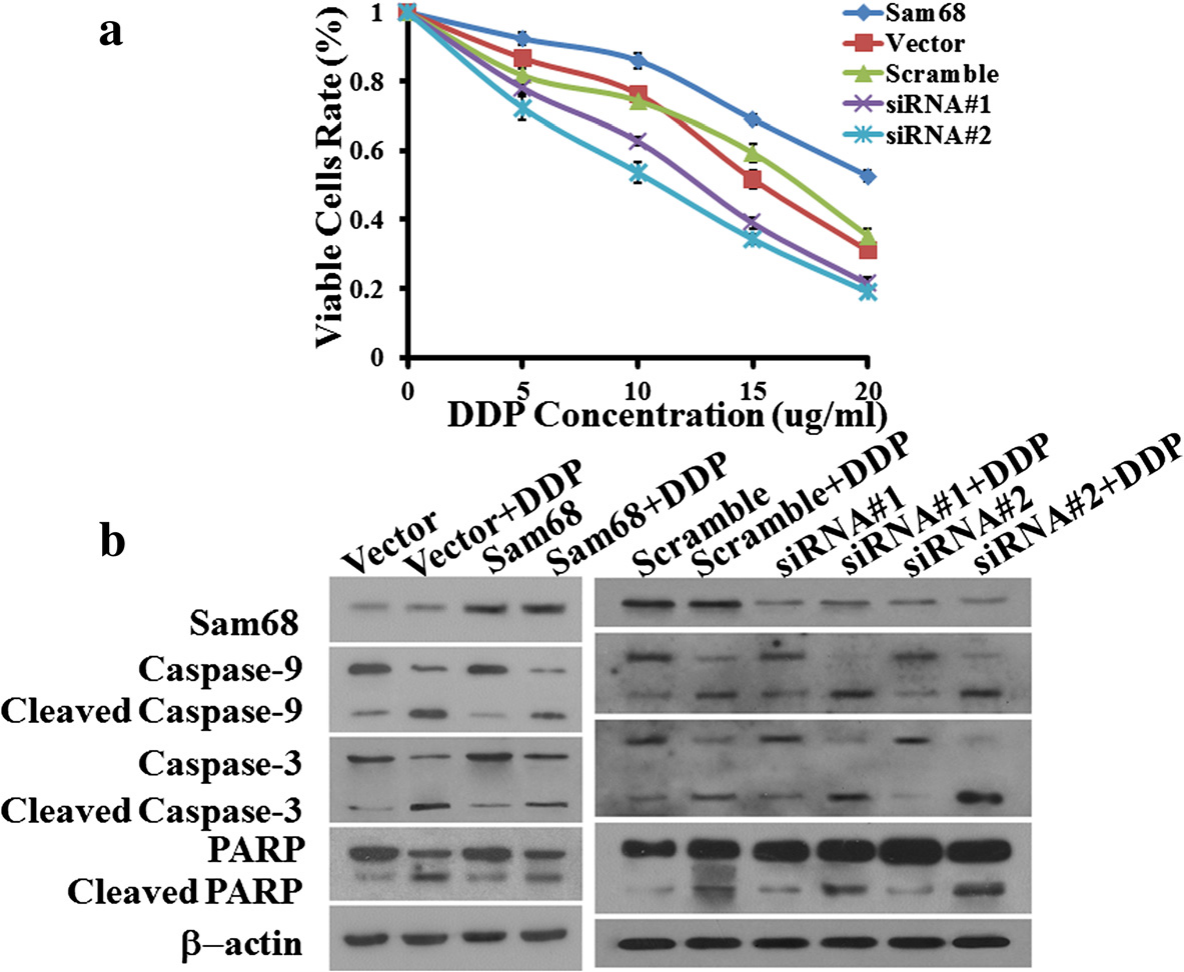
XTT assays demonstrated that the survival rate of SCC-9/Sam68 cells were more resistant to DDP than the vector-control and scramble-control cells. Additionally, SCC-9/siRNA cells were more sensitive to DDP than the vector-control and scramble-control cells (**a**). Sam68 increase activation of caspase 9, caspase 3, and PARP cleavage induced by DDP in cells exhibiting different levels of Sam68 expression (**b**)

To investigate whether Sam68 overexpression contributed to the chemoresistance of OTSCC cells, Sam68 overexpressing cells (SCC-9/Sam68). Sam68 silenced cells (SCC-9/siRNA), and vector-control and scramble-control cells respectively added platinum of different concentration exhibited different survival rate. XTT assays demonstrated that the survival rate of SCC-9/Sam68 cells were more resistant to DDP than the vector-control and scramble-control cells. Additionally, SCC-9/siRNA cells were more sensitive to DDP than the vector-control and scramble-control cells (Fig. 4a).

**Fig. 5:**
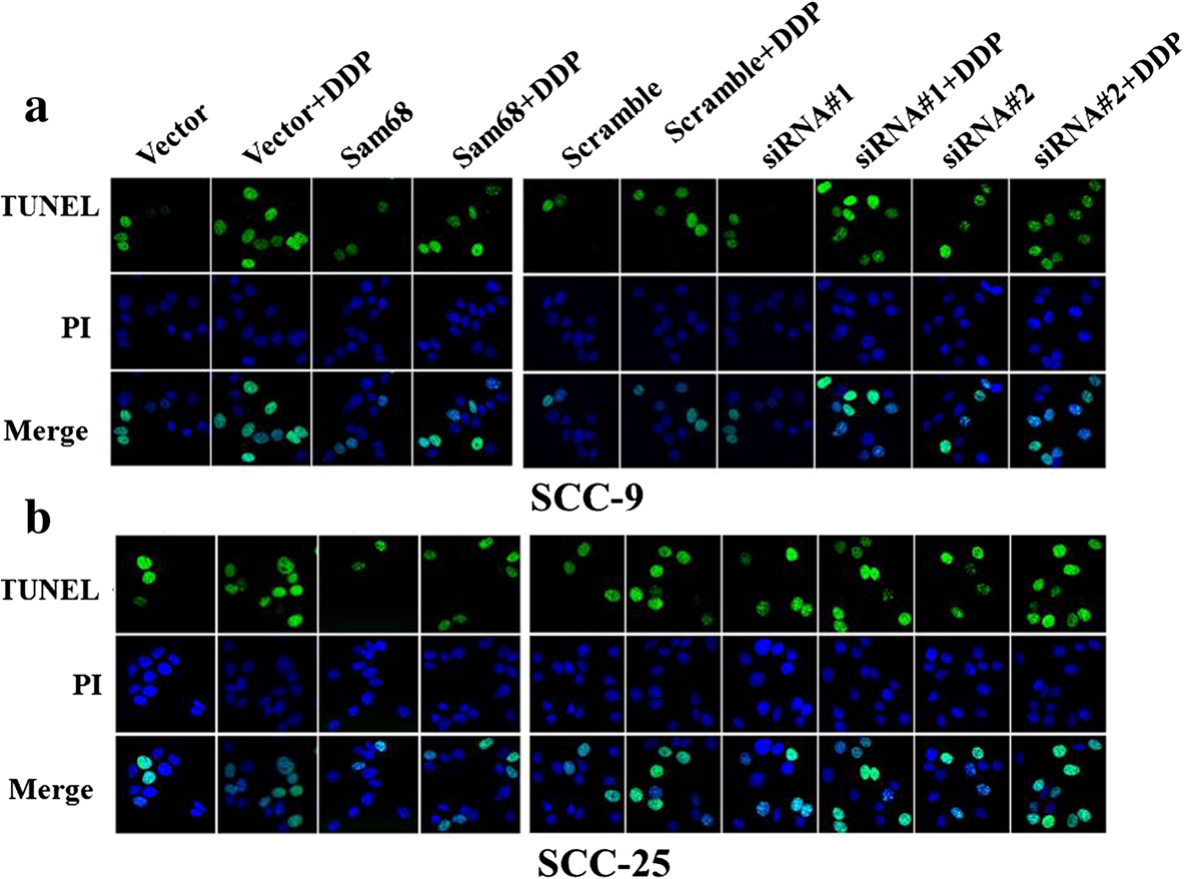
TUNEL assays was carried in two OTSCC cell lines, SCC-9 and SCC-25 (**a**, **b**) to test the anti-resistance and drug-resisstant. The results showed that Sam68 upregulation conferred resistance to OTSCC cells Sam68 upregulation enhanced the resistance of these cells to chemotherapeutics, DDP, and Sam68 downregulation dramatically enhanced the sensitivity of these cells to chemotherapeutics

Furthermore, the effects of Sam68 on apoptosis were confirmed, as observed by increased activation of caspase 9, caspase 3, and PARP cleavage induced by DDP in cells exhibiting different levels of Sam68 expression (Fig. 4b). TUNEL assays showed that Sam68 upregulation conferred resistance to OTSCC cells, and Sam68 downregulation dramatically enhanced the sensitivity of these cells to chemotherapeutics (Fig. 5).

### Sam68 played an important anti-apoptotic role in OTSCC in vivo

**Fig. 6:**
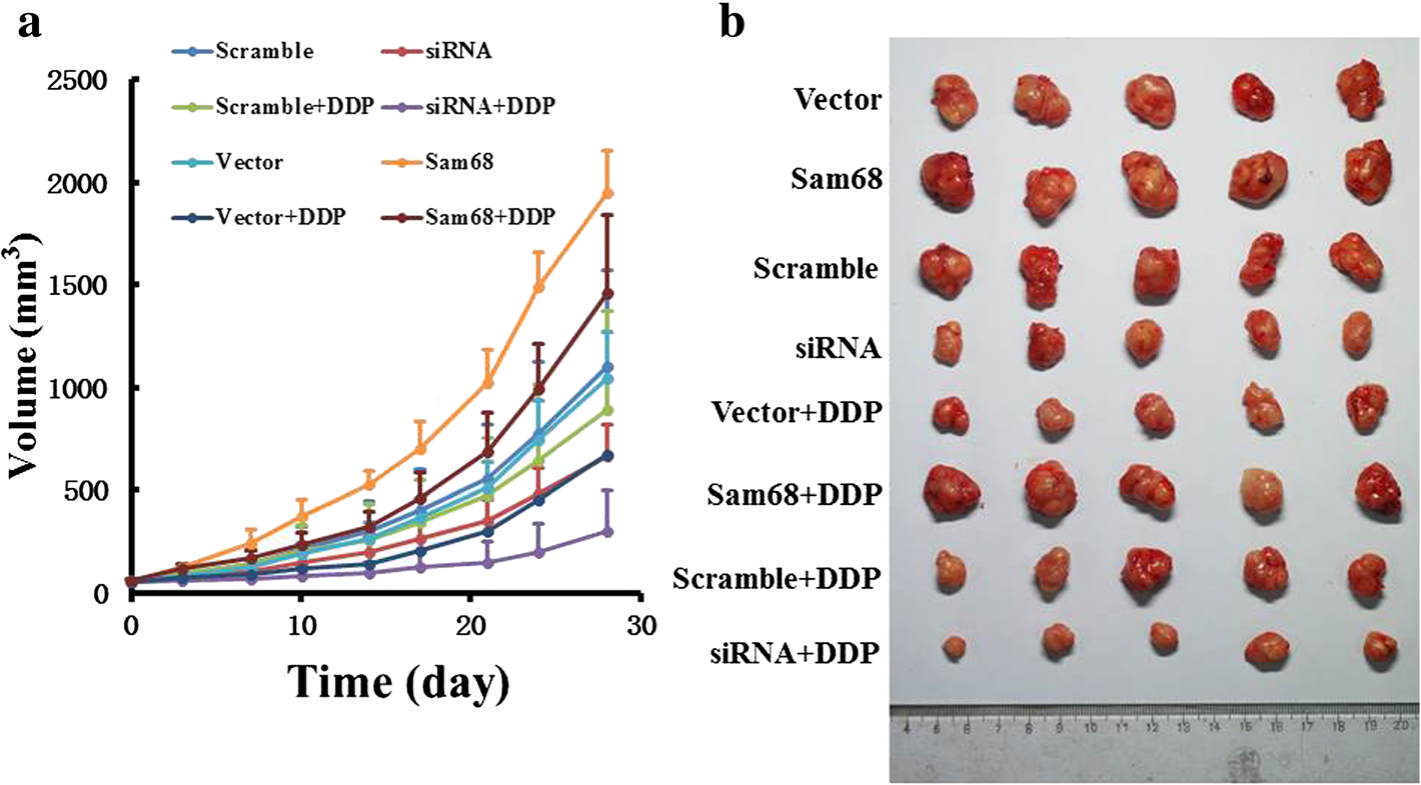
Tumors derived from SCC-9/Sam68 cells grew faster than tumors derived from the vector-control and scramble-control cells. Oppositely the SCC-9/Sam68 shRNA cells grew slowly than tumors derived from the vector-control and scramble-control cells. DDP blocked tumor growth with highest sensitivity in tumors derived from SCC-9/Sam68 shRNA cells, but lowest sensitivity in tumors derived from SCC-9/Sam68 cells (**a**). After 28 days culture, the volumes of tumors formed by SCC-9/Sam68 cells were significantly larger than those of tumors derived from vector-control and scramble-control cells. In contrast, depletion of endogenous Sam68 in SCC-9 cells caused significant inhibition of tumor growth in volume (**b**)

**Fig. 7:**
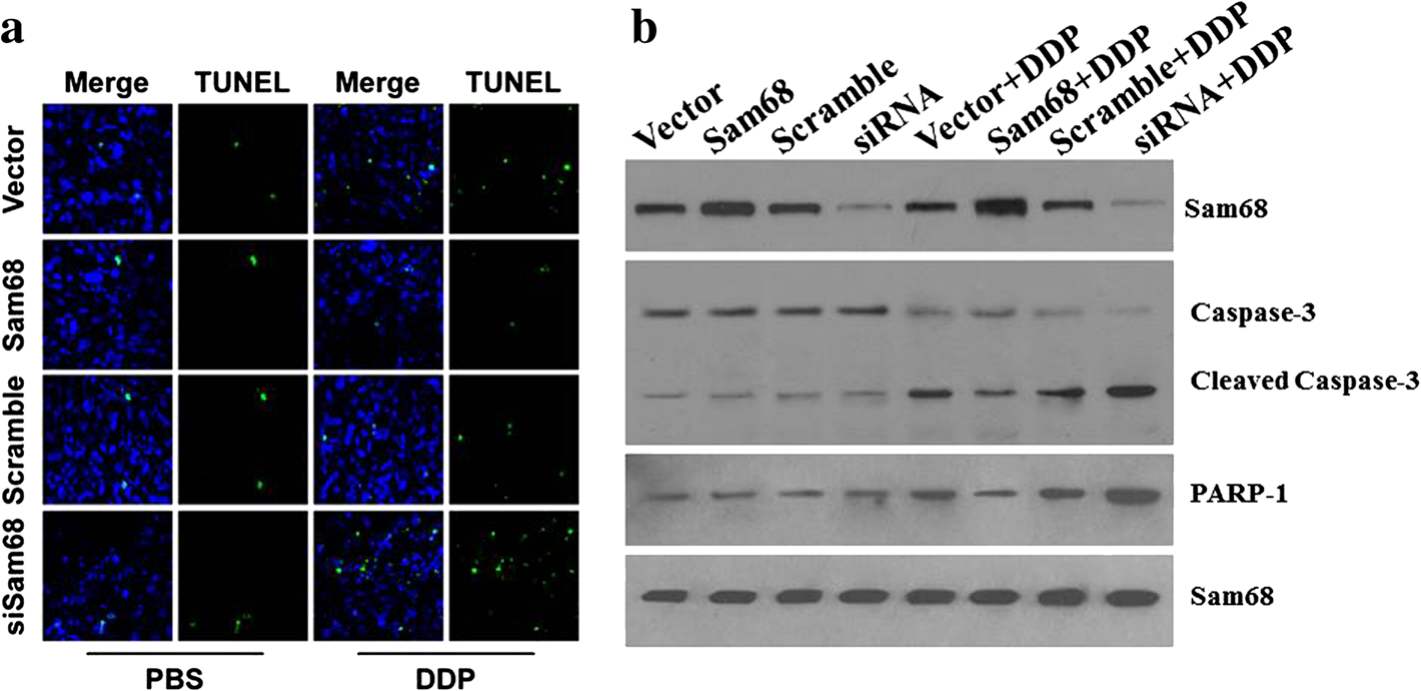
TUNEL assays showed that cells from tumors showing Sam68 upregulation exhibited resistance to DDP, whereas cells derived from tumors showing Sam68 downregulation exhibited dramatically enhanced sensitivity to DDP (**a**). Western blot analysis showed that tumors derived from Sam68-overexpressing cells exhibited increased activation of caspase 9 and PARP cleavage in response to DDP (**b**)

A panel of OTSCC cell lines was constructed to stably express either Sam68 cDNA (SCC-9/Sam68) or Sam68 shRNA (SCC-9/Sam68 shRNA) and inoculated in nude mice. Tumors derived from SCC-9/Sam68 cells grew faster than tumors derived from the SCC-9/Sam68 shRNA cells. DDP blocked tumor growth, with highest sensitivity in tumors derived from SCC-9/Sam68 shRNA cells (Fig. 6a). After 28 days, the volumes and weights of tumors formed by SCC-9/Sam68 cells were significantly larger than those of tumors derived from vector-control and scramble-control cells. In contrast, depletion of endogenous Sam68 in SCC-9 cells caused significant inhibition of tumor growth in terms of both tumor volume and weight (Fig. 6b). TUNEL assays showed that cells from tumors showing Sam68 upregulation exhibited resistance to DDP, whereas cells derived from tumors showing Sam68 downregulation exhibited dramatically enhanced sensitivity to DDP (Fig. 7a).

Western blot analysis showed that tumors derived from Sam68-overexpressing cells exhibited increased activation of caspase 9 and PARP cleavage in response to DDP (Fig. 7b).

## Discussion

OTSCC is one of the most lethal malignancies of the digestive tract in East Asian countries. Many cases are diagnosed after the malignancy has already reached an advanced stage; thus, significant morbidity and mortality are observed due to the disease and sequelae associated with therapeutic management and complications [15, 16]. Though surgery remains the mainstay of treatment for resectable disease, multimodal therapies are necessary to prolong the survival of patients with OTSCC. Empiric clinical trials have defined the standard first-line chemotherapeutic regimens and dosage to achieve optimal therapeutic efficacy with acceptable safety in patients with different types of tumors. The most common chemotherapy drugs used for advanced OTSCC are taxanes (paclitaxel and docetaxel), anthracyclines (adriamycin, epirubicin, and pirarubicin), platinum-based agents (cisplatin and carboplatin), and antimetabolites (e.g., methotrexate and 5-fluorouracil). Platinum-containing compounds, particularly cisplatin, target DNA to form intrastrand and interstrand cross-links; this results in distortion of the DNA helix and apoptotic cell death. However, patients inevitably experience tumor progression or relapses due to the acquisition of drug resistance or the emergence of cell subpopulations genetically refractory to the drugs [17, 18]. A couple of recent studies have reported the role of unfavorable genetic variants and miRNAs in modulating chemoresistance [19–22].

In this study, we demonstrated, for the first time, that Sam68 played a biological role in OTSCC progression and chemotherapy resistance. Additionally, we found that overexpression of Sam68 enhanced anti-apoptotic and drug resistance in OTSCC cell lines treated with DDP. Thus, our findings provide important insights into the role of Sam68 in OTSCC.

Drug resistance is predictable given the common presence of genomic instability in cancers, which can result in the accumulation of multiple genetic aberrations, including those that impact chemotherapy response signaling [23–25]. Evandro Luís Niero et al. comprehensive analysis to find that the resistance of CSCs to currently used chemotherapeutics is a major contributing factor in cancer recurrence and later metastasis development appraise the experimental progress in the study of acquired drug resistance of cancer cells in different models [26]. Ryoichi Fujii et al. pointed that the certain Cox-2 inhibitors may inhibit EMT and lymph node metastasis by restoring E-cadherin expression and down-regulating of CDH-1 in head neck squamous cancer [27]. Zhou et al. revealed miR-223/FBXW7may be a potential strategic approach for reversing the DDP resistance in human gastric carcinoma [28].

In a previous study, we found that Sam68 is overexpressed in OTSCC and that Sam68 is significantly associated with the clinicopathological characteristics and prognosis of patients [13]. NF-kB signaling plays a vital role in cell survival by upregulating gene products that block apoptosis. Additionally, emerging evidence suggests that Sam68 functions as a signaling molecule in multiple signaling pathways [11], including NF-kB activation and apoptosis initiated through the TNF receptor [12]. Consistent with this, our results showed that upregulation of Sam68 significantly inhibited cisplatin-induced apoptosis in OTSCC cells and this effect was associated with induction of the anti-apoptotic proteins caspase-9, caspase-3, and PARP. In contrast, silencing of Sam68 expression significantly enhanced the sensitivity of OTSCC cells to apoptosis induced by cisplatin both in vitro and in vivo.

## Conclusions

In summary, upregulation of Sam68 may reduce cisplatin-induced apoptosis in OTSCC cells; however, the underlying mechanism of acquired chemosensitivity in Sam68-deficient cells is unclear. Further studies are needed to elucidate these mechanisms. Using current therapies, differentiated tumor cells die through apoptosis after platinum-induced DNA damage. Thus, Sam68-mediated inhibition of apoptosis in Sam68-negative tumor cells may facilitate DNA repair, thereby promoting survival.

## Abbreviations

OTSCC, oral tongue squamous cancer cell; Sam68, Src-associated protein in mitosis (68 kDa); TUNEL, Terminal-deoxynucleoitidyl Transferase Mediated Nick End Labeling
